# Citrobacter keratitis: predisposing factors and clinical characteristics

**DOI:** 10.1186/s12348-022-00322-1

**Published:** 2023-01-31

**Authors:** Mohammad Soleimani, Ahmad Masoumi, Seyed Ali Tabatabaei, Mohammad Hossein Zamani

**Affiliations:** Ocular Trauma and Emergency Department, Tehran University of Medical Sciences, 1336616351 Tehran, Iran

**Keywords:** *Citrobacter spp*., Infectious keratitis, Risk factor, Clinical findings

## Abstract

**Purpose:**

To report predisposing factors, clinical presentation, antibiotic sensitivity, and management of Citrobacter-caused infectious keratitis.

**Methods:**

We retrospectively reviewed the medical records of culture-proven cases of Citrobacter keratitis in a tertiary referral center for 8 years (from January 2012 to September 2020). Demographic data of the patients, predisposing factors, and presenting signs were extracted.

**Results:**

Eighteen cases of microbial keratitis due to *Citrobacter spp*. were identified. The median age of the patients was 66 years (range: 10–89, interquartile range : 59–81). Thirteen patients were male and 5 were female. Multiple predisposing factors were identified in all eyes, including ocular surface disease (*n* = 8), previous corneal surgery (*n* = 6), and history of ocular trauma (*n* = 6). Five patients were diabetic. Corrected distance visual acuity (CDVA) of patients was light perception (LP) in 8 patients, hand motion (HM) in 7, counting fingers (CF) at 1 m in 1, and CF at 2 m in 2 patients. Thirteen eyes exhibited hypopyon. An area of corneal thinning was observed in 7 eyes (38.9%). Endophthalmitis due to infectious keratitis developed in one patient. In vitro susceptibility testing confirmed high sensitivity to ceftazidime and aminoglycosides. Medical management consisted primarily of topical amikacin (20 mg/ml) combined with topical cefazoline (50 mg/ml) (72.2%). Surgical tectonic procedures were carried out in 7 eyes (38.9%).

**Conclusion:**

*Citrobacter spp*. is a rare cause of bacterial keratitis.Previous keratoplasty and ocular surface problems are important risk factors. The prognosis is not good and surgical tectonic intervention is required in many cases to resolve the corneal infection.

## Background

Microbial keratitis is an ocular emergency that can lead to a loss of sight and even the eye if it is not treated appropriately. Common clinical signs include conjunctival injection, epithelial defects, corneal ulceration with or without stromal infiltration, and hypopyon. Contact lens wear, extended exposure to topical steroids, dry eye disease, and anterior segment procedures such as corneal transplantation are some of the predisposing factors for the development of infectious keratitis.

*Citrobacter spp.* are gram-negative non-spore-forming bacilli of the family Enterobacteriaceae. They are uncommon causes for urinary and respiratory tract infections [1]. *Citrobacter spp*. are rare etiologic agents in ocular infections [2], and most of the studies focused on Citrobacter keratitis and endophthalmitis are case reports [3, 4]. The literature is scarce regarding the description of clinical features and risk factors of Citrobacter keratitis.

This study aims to investigate the risk factors, clinical characteristics, and antibiotic susceptibility pattern of Citrobacter keratitis that occurred over 8 years in a tertiary eye care center in Iran.

## Materials and methods

This was a retrospective observational case series.The institutional review board of Tehran University of Medical Sciences confirmed that no ethics approval is needed. The clinical and microbiological records of all culture-proven cases of Citrobacter keratitis, who presented to Farabi Eye Hospital from January 2012 to September 2020 were retrospectively reviewed. After slit-lamp examination, all corneal ulcers were scraped using a sterile scalpel blade for Gram stains. Fresh scalpel blades were then used to inoculate in chocolate agar and Sabouraud’s dextrose agar. The culture plates were incubated at 35 °C in carbon dioxide. *Citrobacter spp.* was considered as a causative agent for keratitis if there were discrete colonies of Citrobacter on two solid media or confluent growth of micro-organism was observed along with the site of inoculation. Sulfite indole motility (SIM) medium was used to confirm the growth of *Citrobacter spp.*. Production of H2S and a positive test for motility on SIM agar were considered as evidences of *Citrobacter spp.* growth. Antibiotic susceptibility testing was performed using the disk diffusion method. The following data were collected from patients’ records: age, sex, local and systemic predisposing factors, presenting signs and symptoms including the size of corneal infiltration, size of the epithelial defect, presence of corneal thinning and hypopyon, antibiotic sensitivity, and mode of treatment. Size of corneal epithelial defect was measured by multiplying the longest diameter of the defect by the longest width perpendicular to it.

The patients were admitted if any of the following criteria were present: (1) severe corneal infection according to overall clinical impression (2) presence of corneal thinning or perforation (3) The inability of the patient to instill drops intensively. The initial antibiotic instillation protocol was 1 drop/hour for 24 h. The antibiotic regimen was then modified according to clinical response and antibiotic susceptibility. The indications for adjunctive procedures such as cyanoacrylate glue application or therapeutic penetrating keratoplasty (PKP) were determined by an experienced cornea specialist.

## Results

Within the study period, eighteen microbiologically confirmed Citrobacter keratitis were identified. The age of the patients ranged from 10 to 89 years (median, 66 years, interquartile range: 59–81 ). There were 13(72.2%) male patients and 5(28.8%) female patients. Eleven ulcers developed in the right eye and 7 in the left eye. All patients were admitted and all of them had a combination of one or more risk factors. Thirteen patients in this series had two or more risk factors. During this time, 2522 patients with keratitis were admitted in our center and keratitis caused by Citrobacter spp. comprised 0.7% of admitted patients with infectious keratitis. Risk factors associated with Citrobacter keratitis are reported in Table 1. Preexisting ocular surface disease in the affected eye was the most common predisposing factor (8 of 18 patients, 44.4%); including bullous keratopathy in 4 patients (22.2%), history of herpes simplex virus keratitis in 2 patients (11.1%), neurotropic keratopathy in 1 patient (5.6%) and anesthetic abuse in 1 patient (5.6%). Six cases were diagnosed after previous keratoplasty (33.3%) ; 3 cases (16.7%) had received one or more PKPs and 3 (16.7%) had undergone Descemet stripping automated endothelial keratoplasty (DSAEK). Frequency of topical steroid use in patients with a history of PKP were as follows: one patient did not receive topical steroids, and two patients instilled topical steroids two times a day. In patients who had undergone DSAEK, topical steroids were used as follows: daily in one patient, two times a day in one patient, and three times a day in one patient. Five patients (27.8%) had diabetes mellitus. Six cases (31.6%) presented with a history of ocular trauma. The ocular trauma consisted of trauma with the vegetative matter in 2 cases, bilateral thermal burn in one patient, bilateral alkali burn in one patient, corneal penetrating injury in one case, and corneal foreign body in one case.

**Table 1:** Predisposing factors for development of Citrobacter keratitis (*n* = 18)

Ocular surface disease	8 (44.4%)
Pseudophakic bullous keratopathy	4
History of HSV keratitis	2
Neurotrophic keratopathy	1
Anesthetic abuse	1
Previous corneal surgery	6 (33.3%)
PKP	3
DSAEK	3
History of ocular trauma	6 (33.3%)
Trauma with vegetative matter	2
Thermal burn	1
Alkali burn	1
Corneal penetrating injury	1
Corneal foreign body	1
Diabetes mellitus	5 (27.8%)

Eight patients (44.4%) presented with corrected distance visual acuity (CDVA) of light perception (LP), 7 patients (38.9%) with hand motion (HM) vision, 1 patient (5.5%)with counting fingers (CF) at 1 m, and two patients (11.1%) with CF at 2 m. All patients complained of painful red eyes and decreased vision at presentation. The area of infiltration was small (< 2 mm at its greatest dimension) in 2 eyes, medium (2-6 mm) in 14 eyes, and large (> 6 mm) in 2 eyes. Mean size of corneal epithelial defect at presentation was 19.2 ± 18.6 mm [2]. The location of corneal infiltrate were as follows: Central cornea (8 eyes, 44.4%), paracentral cornea (7 eyes, 38.9%) and peripheral cornea (1 eye, 5.6%). Total corneal infiltration was present in 2 eyes (11.1%). Hypopyon was observed in 13 (72.2%) eyes at initial presentation. Descemet’s membrane folds and endothelial plaques were detected in 7 (38.9%) and 2 (11.1%) eyes, respectively on initial slit-lamp examination. Corneal thinning in the area of infiltration was observed in 7 (38.9%) eyes and the corneal ulcer progressed to perforation in 4 ( 22.2%) patients. Vitreous involvement and endophthalmitis developed in 1 patient (5.6%) after admission. This patient presented with a perforated corneal ulcer in her left eye. Her vision at the presentation was LP. She was diabetic and had a history of cataract surgery and intraocular lens (IOL) exchange in the left eye.

Medical management consisted primarily of topical amikacin (20 mg/ml) combined with topical cefazoline (50 mg/ml) (72.2%) until the specific antibiogram testing was made according to which topical drops were narrowed. Oral doxycycline and vitamin C were prescribed in 77.8% and 66.7% of patients, respectively. In total, 7 patients (38.9%) in the series ultimately required one or more surgical procedures. In the patient who progressed to endophthalmitis, pars plana vitrectomy through a temporary keratoprosthesis associated with the subsequent graft was performed. Three patients underwent therapeutic penetrating keratoplasty. The presence of corneal melt and thinning in two patients necessitated the application of cyanoacrylate glue. Persistent epithelial defect in one patient was treated using amniotic membrane transplantation (AMT).

In vitro testing of *Citrobacter sp.* isolates in this series showed that all were sensitive to gentamicin (18/18), whereas 93.4% isolates were sensitive to amikacin (15/16), 87.5% to ciprofloxacin (14/16), 85.7% to levofloxacin (12/14), and 88.9% to ceftazidime (16/18).

## Discussion

*Citrobacter sp.* belongs to the family Enterobacteriaceae and consists of 13 currently recognized species. *Citrobacter freundi* and *Citrobacter koseri* are mostly associated with human infections [5]. These organisms can be isolated from the intestinal tract of humans. *Citrobacter sp* is often considered an opportunistic pathogen and it causes a broad spectrum of infections related to the urinary tract, respiratory tract, and bloodstream [5]. *Citrobacter sp.* are rare causes for ocular infections [3, 6], and are rarely isolated in patients with bacterial keratitis [2, 7, 8].

Contact lens wear is the most common risk factor for bacterial keratitis in developed countries [9, 10], however, none of the patients in this series were contact lens wearers. A high proportion (44.4%) of patients diagnosed with Citrobacter keratitis suffered from the ocular surface disease. This is comparable to an epidemiologic study of bacterial keratitis from Vancouver, Canada [11], but is much higher compared to the reports of infectious keratitis from northern California (17.7%) [12], and southern Texas (17.6%) [10]. The ocular surface disease impairs the defense mechanisms of the external eye and cornea, moreover, the decreased integrity of the corneal epithelium, persistent epithelial defects, and associated corneal inflammation can contribute to the increased risk of infectious keratitis in these patients [13, 14].

Diabetes mellitus was the most common systemic risk factor in this series. The hyperglycemic state in the diabetic cornea impairs the immunologic defenses of the ocular surface. Moreover, delayed corneal re-epithelialization, and decreased corneal sensation that may progress to neurotrophic corneal ulcers further predisposes the eye to infectious keratitis [15].

It is noteworthy that 6 of 18 patients in our series had a history of corneal transplantation. Epithelial defects, compromised immunity of the ocular surface, suture related problems and graft failure can all contribute to increased risk of graft infection [16]. The most common causative pathogens in infectious keratitis after PKP are gram-positive bacteria, with coagulase-negative staphylococci most commonly isolated. Pseudomonas aeruginosa is the most common inciting gram-negative bacterium in post-PKP infectious keratitis [17]. There is no report of Citrobacter keratitis after PKP in the literature to the best of our knowledge.

None of the eyes in this series were enucleated, however, progression to endophthalmitis necessitated pars plana vitrectomy through a temporary keratoprosthesis in one eye. The incidence of infectious keratitis associated with endophthalmitis is reported to be 0.29% [18]. Malihi et al. found that poor visual acuity, history of ocular surgeries, corneal perforation, topical corticosteroid use, and systemic immunocompromise increases the risk of endophthalmitis in a patient with infectious keratitis [19]. As stated earlier many of these predisposing factors were present in the patient who progressed to endophthalmitis.

Antibiotic resistance is an emerging problem in *Citrobacter sp.*, as in many other bacterial species, and limits the clinician’s armamentarium. In vitro susceptibility testing shows that Citrobacter-caused keratitis may respond well to aminoglycosides. The rate of resistance to levofloxacin (14.7%) and ciprofloxacin (12.5%) was alarmingly higher compared to other reports about antibiotic sensitivity in gram-negative rods [7, 8, 20]. Injudicious use of topical fluoroquinolones primarily for prophylaxis in cataract and refractive surgery might be responsible for this finding. Enhanced efflux of antibiotics and mutations in DNA gyrase subunit B is thought to be responsible for *C.freundi* resistance to fluoroquinolones [21].

The requirement for surgical procedures was 38.9% in this series, which is higher than reports about *Haemophilus influenza* [22] and Moraxella keratitis [23, 24]. This implies the more severe presentation of infectious keratitis due to *Citrobacter spp.* and poorer visual outcomes.

As a tertiary eye care center, many of the patients seeking care in our unit are referred by clinicians who work in an outpatient setting. This may explain the high rate of patients with severe keratitis and who had a history of previous ocular surgeries. The clinicians may have a lower threshold for referral of patients who have undergone corneal surgeries.

The limitations of this study include its small sample size and lack of a control group. The absence of data about follow-up examinations further limits the results of this study. Nevertheless, to the best of our knowledge, this is the largest case series about infectious keratitis due to *Citrobacter spp.*, elucidating the clinical features and risk factors for this devastating infection.

In summary, *Citrobacter spp.* is a rare cause of bacterial keratitis. It comprises 0.7% of all admitted keratitis cases. This study shows that ocular and systemic predisposing factors play an important role in the development of Citrobacter- caused keratitis. Previous keratoplasty and ocular surface problems are important risk factors. Surgical tectonic intervention is required in many cases to resolve the corneal infection.

## Data Availability

The dataset generated during this study is available upon reasonable request.
